# Ethyl 6-chloro-2-[(2-chloro-7,8-dimethyl­quinolin-3-yl)meth­oxy]-4-phenyl­quinoline-3-carboxyl­ate

**DOI:** 10.1107/S1600536810011335

**Published:** 2010-03-27

**Authors:** F. Nawaz Khan, S. Mohana Roopan, Venkatesha R. Hathwar, Mehmet Akkurt

**Affiliations:** a Organic and Medicinal Chemistry Research Laboratory, Organic Chemistry Division, School of Advanced Sciences, VIT University, Vellore 632 014, Tamil Nadu, India; b Solid State and Structural Chemistry Unit, Indian Institute of Science, Bangalore 560 012, Karnataka, India; c Department of Physics, Faculty of Arts and Sciences, Erciyes University, 38039 Kayseri, Turkey

## Abstract

In the title compound, C_30_H_24_Cl_2_N_2_O_3_, the two quinoline ring systems are almost planar [maximum deviations = 0.029 (2) and 0.018 (3) Å] and the dihedral angle between them is 4.17 (8)°. The dihedral angle between the phenyl ring and its attached quinoline ring is 69.06 (13)°. The packing is stabilized by C—H⋯O, C—H⋯N, weak π–π stacking [centroid–centroid distances = 3.7985 (16) and 3.7662 (17) Å] and C—H⋯π inter­actions.

## Related literature

For related structures, see: Khan *et al.* (2009 ▶, 2010*a* ▶,*b* ▶); Roopan *et al.* (2009 ▶). For background to quinolines, see: Roopan & Khan (2009 ▶); Savini *et al.* (2001 ▶).
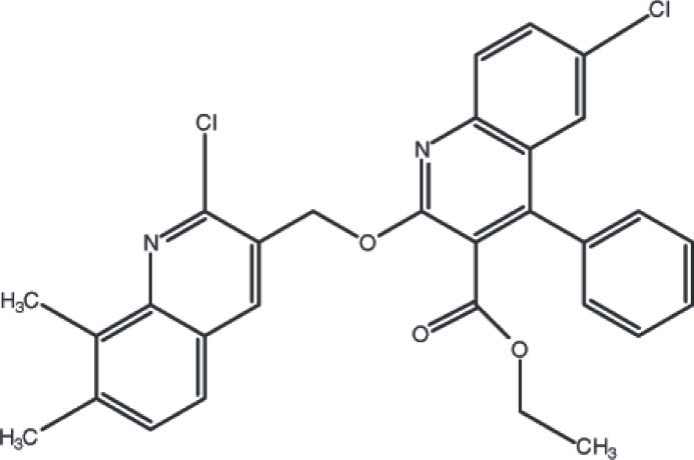


## Experimental

### 

#### Crystal data

C_30_H_24_Cl_2_N_2_O_3_
*M* *_r_* = 531.41
Monoclinic, 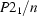

*a* = 8.3187 (5) Å
*b* = 28.0038 (17) Å
*c* = 11.2093 (7) Å
β = 98.721 (6)°
*V* = 2581.1 (3) Å^3^
*Z* = 4
Mo *K*α radiation
μ = 0.29 mm^−1^
*T* = 295 K
0.29 × 0.24 × 0.20 mm

#### Data collection

Oxford Xcalibur Eos (Nova) CCD detector diffractometer
Absorption correction: multi-scan (*CrysAlis PRO RED*; Oxford Diffraction, 2009 ▶) *T*_min_ = 0.921, *T*_max_ = 0.944
25780 measured reflections
4808 independent reflections
1857 reflections with *I* > 2σ(*I*)
*R*_int_ = 0.123

#### Refinement

*R*[*F* ^2^ > 2σ(*F* ^2^)] = 0.048
*wR*(*F* ^2^) = 0.072
*S* = 0.81
4808 reflections
337 parameters
H-atom parameters constrained
Δρ_max_ = 0.18 e Å^−3^
Δρ_min_ = −0.21 e Å^−3^

### 

Data collection: *CrysAlis PRO CCD* (Oxford Diffraction, 2009 ▶); cell refinement: *CrysAlis PRO CCD*; data reduction: *CrysAlis PRO RED* (Oxford Diffraction, 2009 ▶); program(s) used to solve structure: *SHELXS97* (Sheldrick, 2008 ▶); program(s) used to refine structure: *SHELXL97* (Sheldrick, 2008 ▶); molecular graphics: *ORTEP-3 for Windows* (Farrugia, 1997 ▶); software used to prepare material for publication: *WinGX* (Farrugia, 1999 ▶) and *PLATON* (Spek, 2009 ▶).

## Supplementary Material

Crystal structure: contains datablocks global, I. DOI: 10.1107/S1600536810011335/hb5374sup1.cif

Structure factors: contains datablocks I. DOI: 10.1107/S1600536810011335/hb5374Isup2.hkl

Additional supplementary materials:  crystallographic information; 3D view; checkCIF report

## Figures and Tables

**Table 1:** Hydrogen-bond geometry (Å, °) *Cg*2, *Cg*3 and *Cg*5 are the centroids of the N2/C13–C16/C21, C4–C9 and C25–C30 rings, respectively.

*D*—H⋯*A*	*D*—H	H⋯*A*	*D*⋯*A*	*D*—H⋯*A*
C12—H12*A*⋯O2^i^	0.97	2.59	3.364 (3)	137
C26—H26⋯N1^i^	0.93	2.54	3.418 (4)	157
C10—H10*C*⋯*Cg*2^ii^	0.96	2.94	3.753 (3)	143
C12—H12*B*⋯*Cg*3^ii^	0.97	2.82	3.652 (3)	144
C24—H24*B*⋯*Cg*5^iii^	0.96	2.98	3.821 (4)	147

Symmetry codes: (i) 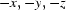; (ii) 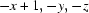; (iii) 
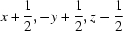
.

**Fig. 1.**
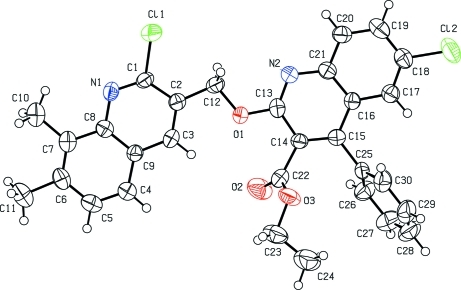
View of (I) with displacement ellipsoids are drawn at the 50% probability level.

**Fig. 2.**
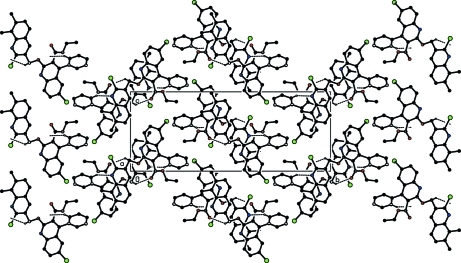
View of the packing diagram and the hydrogen bonding interactions of (I) down the a-axis. All H atoms have been omitted for clarity.

## supplementary crystallographic information

### Comment

A literature search on recent years suggest that there has been sustained interest in the synthesis of quinolines (Roopan *et al.*, 2009; Roopan & Khan, 2009) and are widely used in antimalarial and therapeutic properties. A number of quinoline derivatives are known to possess antitumour, antimicrobial, hypotensive, antileishmanial, anti-HI and anti-inflammatory activities. Application of quinoline derivatives almost spreading in all branch of medicinal chemistry. The chemistry of quinolinylquinoline derivatives contuse to draw attention of synthetic organic chemist due to their varied biological activities. Prompted by recent literature observations (Savini *et al.*, 2001) and as a part of our search for bio-active quinoline derivatives, we undertook the synthesis of quinolinylquinoline.

In the title molecule (I), Fig. 1, there are two quinoline ring systems (N1/C1–C9) and (N2/C13–C21) and they are almost planar, with maximum deviations of 0.029 (2) Å for atom N1 and -0.018 (3) Å for atom C17, respectively. The quinoline systems (N1/C1–C9) and (N2/C13–C21 make a dihedral angle of 4.17 (8)° with each other and, dihedral angles of 68.68 (13)° and 69.06 (13)°, respectively, with the phenyl ring (C25–C30).

In the title molecule, there are weak intramolecular C—H···O and C—H···N interactions (Table 1). The crystal packing is stabilized by weak π-π interactions [Cg1···Cg1(1-x, -y, -z) = 3.7985 (16) and Cg3···Cg4(-x, -y, -z) = 3.7662 (17); where Cg1, Cg3 and Cg4 are the centroids of the N1/C1–C3/C8/C9, C4–C9 and C16–C21 rings, respectively]. In the crystal structure, there are also some C—H···π interactions (Table 1). A view of the packing diagram down the a-axis is shown in Fig. 2.

### Experimental

To a well-mixed solution of ethyl6-chloro-1,2-dihydro-2-oxo-4-phenyl quinoline-3-carboxylate (327 mg, 1 mmol, in 2 ml of DMF), KO^t^Bu (112 mg, 1 mmol, in 10 ml THF) and 2-chloro-3-(chloromethyl)-7,8-dimethylquinoline (239 mg, 1 mmol) were added and the resulting mixture was refluxed at 343 K for 1 h. Completion of the reaction was monitored by TLC. After that, excess solvent was removed under reduced pressure. The residue was mixed well with crushed ice. Separated solid was filtered, dried in air and then re-crystallized with chloroform.  Colourless blocks of (I) were grown by solvent evaporation from a solution of the compound in acetone.

### Refinement

All H atoms were placed in geometrically idealized positions and constrained to ride on their parent atoms, with C—H = 0.93-0.97 Å and U_iso_(H) = 1.2 or 1.5 U_eq_(C).

### Crystal data

**Table:**

C_30_H_24_Cl_2_N_2_O_3_	*F*(000) = 1104
*M**_r_* = 531.41	*D*_x_ = 1.367 Mg m^−^^3^
Monoclinic, *P*2_1_/*n*	Mo *K*α radiation, λ = 0.71073 Å
Hall symbol: -P 2yn	Cell parameters from 985 reflections
*a* = 8.3187 (5) Å	θ = 2.6–25.5°
*b* = 28.0038 (17) Å	µ = 0.29 mm^−^^1^
*c* = 11.2093 (7) Å	*T* = 295 K
β = 98.721 (6)°	Block, colourless
*V* = 2581.1 (3) Å^3^	0.29 × 0.24 × 0.20 mm
*Z* = 4	

### Data collection

**Table:**

Oxford Xcalibur Eos (Nova) CCD detector diffractometer	4808 independent reflections
Radiation source: Enhance (Mo) X-ray Source	1857 reflections with *I* > 2σ(*I*)
graphite	*R*_int_ = 0.123
ω scans	θ_max_ = 25.5°, θ_min_ = 2.6°
Absorption correction: multi-scan (*CrysAlis PRO* RED; Oxford Diffraction, 2009)	*h* = −10→10
*T*_min_ = 0.921, *T*_max_ = 0.944	*k* = −33→33
25780 measured reflections	*l* = −13→13

### Refinement

**Table:**

Refinement on *F*^2^	Primary atom site location: structure-invariant direct methods
Least-squares matrix: full	Secondary atom site location: difference Fourier map
*R*[*F*^2^ > 2σ(*F*^2^)] = 0.048	Hydrogen site location: inferred from neighbouring sites
*wR*(*F*^2^) = 0.072	H-atom parameters constrained
*S* = 0.81	*w* = 1/[σ^2^(*F*_o_^2^) + (0.0132*P*)^2^] where *P* = (*F*_o_^2^ + 2*F*_c_^2^)/3
4808 reflections	(Δ/σ)_max_ < 0.001
337 parameters	Δρ_max_ = 0.18 e Å^−^^3^
0 restraints	Δρ_min_ = −0.21 e Å^−^^3^

### Special details

**Table:**

Geometry. Bond distances, angles etc. have been calculated using the rounded fractional coordinates. All su's are estimated from the variances of the (full) variance-covariance matrix. The cell esds are taken into account in the estimation of distances, angles and torsion angles
Refinement. Refinement on *F*^2^ for ALL reflections except those flagged by the user for potential systematic errors. Weighted *R*-factors wR and all goodnesses of fit *S* are based on *F*^2^, conventional *R*-factors *R* are based on *F*, with *F* set to zero for negative *F*^2^. The observed criterion of *F*^2^ > σ(*F*^2^) is used only for calculating -*R*-factor-obs etc. and is not relevant to the choice of reflections for refinement. *R*-factors based on *F*^2^ are statistically about twice as large as those based on *F*, and *R*-factors based on ALL data will be even larger.

### Fractional atomic coordinates and isotropic or equivalent isotropic displacement parameters (Å^2^)

**Table:**

	*x*	*y*	*z*	*U*_iso_*/*U*_eq_	
Cl1	0.41574 (10)	−0.09830 (3)	0.17492 (7)	0.0664 (3)	
Cl2	−0.16048 (10)	0.18075 (3)	0.62865 (7)	0.0769 (4)	
O1	0.1593 (2)	0.03957 (7)	0.09664 (16)	0.0491 (8)	
O2	−0.0023 (3)	0.10440 (8)	−0.08652 (19)	0.0761 (10)	
O3	0.1613 (2)	0.16113 (8)	0.00331 (17)	0.0582 (9)	
N1	0.4330 (3)	−0.09180 (8)	−0.0534 (2)	0.0431 (10)	
N2	0.0969 (3)	0.05269 (8)	0.2868 (2)	0.0408 (10)	
C1	0.3783 (3)	−0.07019 (10)	0.0336 (3)	0.0414 (11)	
C2	0.2954 (3)	−0.02581 (10)	0.0283 (3)	0.0379 (12)	
C3	0.2746 (3)	−0.00415 (10)	−0.0811 (3)	0.0417 (11)	
C4	0.3142 (3)	−0.00413 (11)	−0.2960 (3)	0.0504 (12)	
C5	0.3682 (4)	−0.02691 (12)	−0.3888 (3)	0.0546 (14)	
C6	0.4432 (3)	−0.07216 (12)	−0.3737 (3)	0.0480 (12)	
C7	0.4630 (3)	−0.09424 (11)	−0.2636 (3)	0.0432 (11)	
C8	0.4087 (3)	−0.07039 (11)	−0.1658 (3)	0.0395 (12)	
C9	0.3322 (3)	−0.02535 (10)	−0.1802 (3)	0.0378 (12)	
C10	0.5389 (3)	−0.14318 (10)	−0.2462 (3)	0.0614 (14)	
C11	0.4988 (3)	−0.09507 (11)	−0.4838 (2)	0.0705 (14)	
C12	0.2312 (3)	−0.00526 (9)	0.1354 (3)	0.0453 (12)	
C13	0.0961 (3)	0.06702 (11)	0.1771 (3)	0.0410 (12)	
C14	0.0399 (3)	0.11247 (10)	0.1293 (3)	0.0372 (12)	
C15	−0.0212 (3)	0.14355 (10)	0.2034 (3)	0.0358 (11)	
C16	−0.0267 (3)	0.12959 (11)	0.3255 (3)	0.0357 (11)	
C17	−0.0888 (3)	0.15872 (10)	0.4103 (3)	0.0436 (12)	
C18	−0.0858 (3)	0.14366 (12)	0.5255 (3)	0.0491 (12)	
C19	−0.0249 (3)	0.09918 (12)	0.5634 (3)	0.0548 (14)	
C20	0.0334 (3)	0.06967 (11)	0.4830 (3)	0.0483 (12)	
C21	0.0335 (3)	0.08365 (11)	0.3622 (3)	0.0403 (12)	
C22	0.0606 (4)	0.12451 (12)	0.0010 (3)	0.0479 (14)	
C23	0.1936 (4)	0.18050 (13)	−0.1109 (3)	0.0786 (17)	
C24	0.1264 (5)	0.22927 (12)	−0.1233 (3)	0.117 (2)	
C25	−0.0804 (4)	0.19251 (10)	0.1607 (3)	0.0383 (12)	
C26	−0.2210 (4)	0.19679 (11)	0.0785 (3)	0.0546 (12)	
C27	−0.2764 (4)	0.24215 (13)	0.0418 (3)	0.0706 (17)	
C28	−0.1930 (5)	0.28234 (12)	0.0844 (3)	0.0725 (17)	
C29	−0.0541 (4)	0.27775 (12)	0.1640 (3)	0.0652 (16)	
C30	0.0047 (3)	0.23285 (12)	0.2034 (3)	0.0548 (14)	
H3	0.22160	0.02510	−0.09080	0.0500*	
H4	0.26490	0.02560	−0.30850	0.0610*	
H5	0.35560	−0.01240	−0.46430	0.0660*	
H10A	0.46760	−0.16630	−0.28950	0.0920*	
H10B	0.55640	−0.15100	−0.16180	0.0920*	
H10C	0.64110	−0.14330	−0.27610	0.0920*	
H11A	0.61550	−0.09610	−0.47270	0.1060*	
H11B	0.45970	−0.07660	−0.55440	0.1060*	
H11C	0.45630	−0.12690	−0.49370	0.1060*	
H12A	0.15080	−0.02640	0.16140	0.0550*	
H12B	0.31880	−0.00070	0.20220	0.0550*	
H17	−0.13210	0.18850	0.38710	0.0530*	
H19	−0.02390	0.08950	0.64290	0.0660*	
H20	0.07380	0.03980	0.50840	0.0580*	
H23A	0.14290	0.16070	−0.17710	0.0940*	
H23B	0.30980	0.18120	−0.11270	0.0940*	
H24A	0.01300	0.22850	−0.11590	0.1760*	
H24B	0.13930	0.24190	−0.20100	0.1760*	
H24C	0.18330	0.24920	−0.06120	0.1760*	
H26	−0.27790	0.16970	0.04800	0.0650*	
H27	−0.37200	0.24520	−0.01270	0.0850*	
H28	−0.23130	0.31240	0.05910	0.0870*	
H29	0.00310	0.30500	0.19280	0.0780*	
H30	0.10030	0.23020	0.25800	0.0660*	

### Atomic displacement parameters (Å^2^)

**Table:**

	*U*^11^	*U*^22^	*U*^33^	*U*^12^	*U*^13^	*U*^23^
Cl1	0.0992 (7)	0.0524 (5)	0.0464 (6)	0.0156 (5)	0.0069 (5)	0.0096 (5)
Cl2	0.0844 (6)	0.0995 (8)	0.0504 (6)	0.0134 (6)	0.0214 (5)	−0.0159 (6)
O1	0.0695 (15)	0.0410 (13)	0.0387 (14)	0.0177 (12)	0.0145 (11)	0.0021 (12)
O2	0.111 (2)	0.0710 (17)	0.0426 (17)	−0.0241 (16)	0.0002 (14)	−0.0091 (14)
O3	0.0746 (16)	0.0637 (16)	0.0393 (15)	−0.0123 (14)	0.0185 (12)	0.0066 (13)
N1	0.0479 (17)	0.0351 (16)	0.0463 (18)	0.0037 (13)	0.0073 (14)	−0.0069 (15)
N2	0.0441 (16)	0.0414 (17)	0.0374 (17)	0.0010 (13)	0.0080 (14)	0.0012 (15)
C1	0.052 (2)	0.0300 (19)	0.040 (2)	0.0020 (17)	0.0001 (17)	0.0026 (17)
C2	0.040 (2)	0.035 (2)	0.037 (2)	−0.0036 (16)	0.0005 (16)	−0.0030 (17)
C3	0.042 (2)	0.0336 (19)	0.049 (2)	0.0054 (16)	0.0058 (17)	−0.0024 (18)
C4	0.058 (2)	0.050 (2)	0.044 (2)	0.0052 (18)	0.0101 (18)	0.002 (2)
C5	0.062 (2)	0.062 (3)	0.040 (2)	−0.003 (2)	0.0089 (18)	0.006 (2)
C6	0.041 (2)	0.058 (2)	0.046 (2)	−0.0081 (19)	0.0101 (18)	−0.013 (2)
C7	0.0334 (19)	0.045 (2)	0.050 (2)	−0.0063 (17)	0.0027 (17)	−0.012 (2)
C8	0.039 (2)	0.041 (2)	0.038 (2)	−0.0057 (17)	0.0039 (16)	−0.0024 (18)
C9	0.042 (2)	0.034 (2)	0.037 (2)	−0.0003 (17)	0.0049 (16)	−0.0016 (18)
C10	0.058 (2)	0.060 (2)	0.068 (3)	0.004 (2)	0.0154 (18)	−0.013 (2)
C11	0.075 (2)	0.085 (3)	0.055 (2)	−0.010 (2)	0.0216 (19)	−0.015 (2)
C12	0.057 (2)	0.035 (2)	0.043 (2)	0.0024 (17)	0.0045 (17)	0.0048 (17)
C13	0.038 (2)	0.044 (2)	0.041 (2)	0.0028 (18)	0.0056 (17)	−0.008 (2)
C14	0.043 (2)	0.037 (2)	0.031 (2)	−0.0002 (17)	0.0039 (16)	0.0036 (17)
C15	0.0320 (19)	0.036 (2)	0.039 (2)	−0.0060 (16)	0.0044 (16)	0.0016 (17)
C16	0.0286 (18)	0.039 (2)	0.040 (2)	−0.0006 (16)	0.0073 (16)	−0.0008 (17)
C17	0.039 (2)	0.048 (2)	0.044 (2)	0.0028 (17)	0.0067 (17)	0.0008 (19)
C18	0.043 (2)	0.061 (2)	0.044 (2)	0.0015 (19)	0.0092 (17)	−0.004 (2)
C19	0.051 (2)	0.076 (3)	0.038 (2)	−0.002 (2)	0.0085 (17)	−0.001 (2)
C20	0.052 (2)	0.050 (2)	0.043 (2)	0.0035 (18)	0.0079 (18)	0.0073 (19)
C21	0.038 (2)	0.045 (2)	0.037 (2)	−0.0021 (17)	0.0030 (16)	−0.0001 (18)
C22	0.053 (2)	0.037 (2)	0.054 (3)	0.0071 (19)	0.009 (2)	0.002 (2)
C23	0.105 (3)	0.080 (3)	0.057 (3)	−0.009 (3)	0.032 (2)	0.016 (2)
C24	0.221 (5)	0.068 (3)	0.073 (3)	−0.017 (3)	0.054 (3)	0.007 (3)
C25	0.046 (2)	0.034 (2)	0.037 (2)	0.0016 (18)	0.0133 (16)	−0.0047 (17)
C26	0.061 (2)	0.042 (2)	0.058 (2)	−0.0007 (19)	0.0005 (19)	0.0012 (19)
C27	0.068 (3)	0.063 (3)	0.078 (3)	0.009 (2)	0.002 (2)	0.019 (2)
C28	0.080 (3)	0.044 (3)	0.101 (3)	0.012 (2)	0.038 (2)	0.024 (2)
C29	0.076 (3)	0.038 (2)	0.090 (3)	−0.013 (2)	0.040 (2)	−0.007 (2)
C30	0.055 (2)	0.048 (2)	0.063 (3)	−0.002 (2)	0.0141 (18)	−0.010 (2)

### Geometric parameters (Å, °)

**Table:**

Cl1—C1	1.754 (3)	C20—C21	1.410 (5)
Cl2—C18	1.738 (3)	C23—C24	1.474 (5)
O1—C12	1.430 (3)	C25—C26	1.380 (5)
O1—C13	1.351 (4)	C25—C30	1.380 (4)
O2—C22	1.182 (4)	C26—C27	1.392 (5)
O3—C22	1.322 (4)	C27—C28	1.370 (5)
O3—C23	1.453 (4)	C28—C29	1.355 (5)
N1—C1	1.288 (4)	C29—C30	1.396 (5)
N1—C8	1.382 (4)	C3—H3	0.9300
N2—C13	1.293 (4)	C4—H4	0.9300
N2—C21	1.371 (4)	C5—H5	0.9300
C1—C2	1.418 (4)	C10—H10A	0.9600
C2—C3	1.356 (5)	C10—H10B	0.9600
C2—C12	1.501 (4)	C10—H10C	0.9600
C3—C9	1.406 (4)	C11—H11A	0.9600
C4—C5	1.354 (5)	C11—H11B	0.9600
C4—C9	1.415 (5)	C11—H11C	0.9600
C5—C6	1.411 (5)	C12—H12A	0.9700
C6—C7	1.368 (5)	C12—H12B	0.9700
C6—C11	1.524 (4)	C17—H17	0.9300
C7—C8	1.415 (4)	C19—H19	0.9300
C7—C10	1.509 (4)	C20—H20	0.9300
C8—C9	1.411 (4)	C23—H23A	0.9700
C13—C14	1.432 (4)	C23—H23B	0.9700
C14—C15	1.354 (4)	C24—H24A	0.9600
C14—C22	1.512 (5)	C24—H24B	0.9600
C15—C16	1.431 (5)	C24—H24C	0.9600
C15—C25	1.510 (4)	C26—H26	0.9300
C16—C17	1.409 (4)	C27—H27	0.9300
C16—C21	1.419 (4)	C28—H28	0.9300
C17—C18	1.355 (5)	C29—H29	0.9300
C18—C19	1.387 (5)	C30—H30	0.9300
C19—C20	1.365 (4)		
			
C12—O1—C13	119.0 (2)	C26—C27—C28	121.3 (3)
C22—O3—C23	118.3 (2)	C27—C28—C29	119.2 (3)
C1—N1—C8	117.8 (2)	C28—C29—C30	121.1 (3)
C13—N2—C21	116.3 (3)	C25—C30—C29	119.4 (3)
Cl1—C1—N1	116.0 (2)	C2—C3—H3	120.00
Cl1—C1—C2	116.7 (2)	C9—C3—H3	119.00
N1—C1—C2	127.3 (3)	C5—C4—H4	120.00
C1—C2—C3	115.1 (3)	C9—C4—H4	120.00
C1—C2—C12	122.2 (3)	C4—C5—H5	119.00
C3—C2—C12	122.7 (3)	C6—C5—H5	119.00
C2—C3—C9	120.9 (3)	C7—C10—H10A	109.00
C5—C4—C9	120.4 (3)	C7—C10—H10B	109.00
C4—C5—C6	121.5 (3)	C7—C10—H10C	109.00
C5—C6—C7	120.3 (3)	H10A—C10—H10B	110.00
C5—C6—C11	117.6 (3)	H10A—C10—H10C	109.00
C7—C6—C11	122.1 (3)	H10B—C10—H10C	109.00
C6—C7—C8	118.5 (3)	C6—C11—H11A	109.00
C6—C7—C10	121.2 (3)	C6—C11—H11B	110.00
C8—C7—C10	120.3 (3)	C6—C11—H11C	109.00
N1—C8—C7	118.8 (3)	H11A—C11—H11B	109.00
N1—C8—C9	119.6 (3)	H11A—C11—H11C	110.00
C7—C8—C9	121.7 (3)	H11B—C11—H11C	109.00
C3—C9—C4	123.1 (3)	O1—C12—H12A	111.00
C3—C9—C8	119.3 (3)	O1—C12—H12B	110.00
C4—C9—C8	117.6 (3)	C2—C12—H12A	111.00
O1—C12—C2	106.1 (2)	C2—C12—H12B	111.00
O1—C13—N2	120.8 (3)	H12A—C12—H12B	109.00
O1—C13—C14	113.2 (3)	C16—C17—H17	120.00
N2—C13—C14	125.9 (3)	C18—C17—H17	120.00
C13—C14—C15	118.3 (3)	C18—C19—H19	120.00
C13—C14—C22	118.5 (3)	C20—C19—H19	120.00
C15—C14—C22	123.2 (3)	C19—C20—H20	119.00
C14—C15—C16	118.7 (3)	C21—C20—H20	119.00
C14—C15—C25	121.6 (3)	O3—C23—H23A	110.00
C16—C15—C25	119.7 (3)	O3—C23—H23B	110.00
C15—C16—C17	123.7 (3)	C24—C23—H23A	110.00
C15—C16—C21	117.7 (3)	C24—C23—H23B	110.00
C17—C16—C21	118.6 (3)	H23A—C23—H23B	108.00
C16—C17—C18	120.5 (3)	C23—C24—H24A	110.00
Cl2—C18—C17	119.4 (2)	C23—C24—H24B	109.00
Cl2—C18—C19	118.9 (3)	C23—C24—H24C	109.00
C17—C18—C19	121.6 (3)	H24A—C24—H24B	109.00
C18—C19—C20	119.4 (3)	H24A—C24—H24C	109.00
C19—C20—C21	121.2 (3)	H24B—C24—H24C	109.00
N2—C21—C16	123.1 (3)	C25—C26—H26	120.00
N2—C21—C20	118.2 (3)	C27—C26—H26	121.00
C16—C21—C20	118.6 (3)	C26—C27—H27	119.00
O2—C22—O3	125.8 (3)	C28—C27—H27	119.00
O2—C22—C14	125.7 (3)	C27—C28—H28	120.00
O3—C22—C14	108.5 (3)	C29—C28—H28	120.00
O3—C23—C24	108.1 (3)	C28—C29—H29	119.00
C15—C25—C26	119.6 (3)	C30—C29—H29	119.00
C15—C25—C30	120.5 (3)	C25—C30—H30	120.00
C26—C25—C30	119.9 (3)	C29—C30—H30	120.00
C25—C26—C27	119.1 (3)		
			
C13—O1—C12—C2	−177.7 (2)	O1—C13—C14—C15	−178.0 (2)
C12—O1—C13—N2	−1.6 (4)	O1—C13—C14—C22	−1.4 (4)
C12—O1—C13—C14	175.3 (2)	N2—C13—C14—C15	−1.3 (4)
C23—O3—C22—O2	3.1 (5)	N2—C13—C14—C22	175.2 (3)
C23—O3—C22—C14	−177.2 (2)	C13—C14—C15—C16	0.0 (4)
C22—O3—C23—C24	113.9 (3)	C13—C14—C15—C25	179.0 (3)
C8—N1—C1—Cl1	−178.8 (2)	C22—C14—C15—C16	−176.3 (3)
C8—N1—C1—C2	−0.6 (4)	C22—C14—C15—C25	2.6 (4)
C1—N1—C8—C7	−177.9 (3)	C13—C14—C22—O2	64.1 (4)
C1—N1—C8—C9	2.6 (4)	C13—C14—C22—O3	−115.7 (3)
C21—N2—C13—O1	178.5 (2)	C15—C14—C22—O2	−119.6 (4)
C21—N2—C13—C14	2.0 (4)	C15—C14—C22—O3	60.7 (4)
C13—N2—C21—C16	−1.6 (4)	C14—C15—C16—C17	−179.4 (3)
C13—N2—C21—C20	−179.4 (3)	C14—C15—C16—C21	0.3 (4)
Cl1—C1—C2—C3	177.3 (2)	C25—C15—C16—C17	1.6 (4)
Cl1—C1—C2—C12	−4.5 (3)	C25—C15—C16—C21	−178.7 (3)
N1—C1—C2—C3	−0.9 (4)	C14—C15—C25—C26	70.0 (4)
N1—C1—C2—C12	177.4 (3)	C14—C15—C25—C30	−110.1 (4)
C1—C2—C3—C9	0.2 (4)	C16—C15—C25—C26	−111.1 (3)
C12—C2—C3—C9	−178.0 (2)	C16—C15—C25—C30	68.8 (4)
C1—C2—C12—O1	178.9 (2)	C15—C16—C17—C18	−178.1 (3)
C3—C2—C12—O1	−3.1 (3)	C21—C16—C17—C18	2.2 (4)
C2—C3—C9—C4	−179.7 (3)	C15—C16—C21—N2	0.5 (4)
C2—C3—C9—C8	1.7 (4)	C15—C16—C21—C20	178.3 (2)
C9—C4—C5—C6	0.1 (5)	C17—C16—C21—N2	−179.8 (3)
C5—C4—C9—C3	−178.1 (3)	C17—C16—C21—C20	−2.0 (4)
C5—C4—C9—C8	0.4 (4)	C16—C17—C18—Cl2	179.0 (2)
C4—C5—C6—C7	0.1 (5)	C16—C17—C18—C19	−1.2 (4)
C4—C5—C6—C11	179.6 (3)	Cl2—C18—C19—C20	179.7 (2)
C5—C6—C7—C8	−0.8 (4)	C17—C18—C19—C20	−0.1 (4)
C5—C6—C7—C10	178.4 (3)	C18—C19—C20—C21	0.3 (4)
C11—C6—C7—C8	179.7 (2)	C19—C20—C21—N2	178.7 (3)
C11—C6—C7—C10	−1.1 (4)	C19—C20—C21—C16	0.8 (4)
C6—C7—C8—N1	−178.0 (3)	C15—C25—C26—C27	178.6 (3)
C6—C7—C8—C9	1.5 (4)	C30—C25—C26—C27	−1.3 (5)
C10—C7—C8—N1	2.8 (4)	C15—C25—C30—C29	−178.9 (3)
C10—C7—C8—C9	−177.8 (2)	C26—C25—C30—C29	0.9 (5)
N1—C8—C9—C3	−3.2 (4)	C25—C26—C27—C28	0.9 (5)
N1—C8—C9—C4	178.2 (2)	C26—C27—C28—C29	−0.1 (5)
C7—C8—C9—C3	177.4 (3)	C27—C28—C29—C30	−0.2 (5)
C7—C8—C9—C4	−1.3 (4)	C28—C29—C30—C25	−0.2 (5)

### Hydrogen-bond geometry (Å, °)

**Table:**

Cg2, Cg3 and Cg5 are the centroids of the N2/C13–C16/C21, C4–C9 and C25–C30 rings, respectively.

**Table:**

*D*—H···*A*	*D*—H	H···*A*	*D*···*A*	*D*—H···*A*
C12—H12A···O2^i^	0.97	2.59	3.364 (3)	137
C26—H26···N1^i^	0.93	2.54	3.418 (4)	157
C10—H10C···Cg2^ii^	0.96	2.94	3.753 (3)	143
C12—H12B···Cg3^ii^	0.97	2.82	3.652 (3)	144
C24—H24B···Cg5^iii^	0.96	2.98	3.821 (4)	147

Symmetry codes: (i) −*x*, −*y*, −*z*; (ii) −*x*+1, −*y*, −*z*; (iii) *x*+1/2, −*y*+1/2, *z*−1/2.
